# Following the footprints of polymorphic inversions on SNP data: from detection to association tests

**DOI:** 10.1093/nar/gkv073

**Published:** 2015-02-11

**Authors:** Alejandro Cáceres, Juan R. González

**Affiliations:** 1 Center for Research in Environmental Epidemiology (CREAL), Doctor Aiguader 88, Barcelona 08003, Spain; 2 IMIM (Hospital del Mar Research Institute), Doctor Aiguader 88, Barcelona 08003, Spain; 3 Centro de Investigacion Biomedica en Red en Epidemiologia y Salud Publica (CIBERESP), Barcelona 08036, Spain; 4 Department of Mathematics, Universitat Autonoma de Barcelona (UAB), Barcelona 08193, Spain

## Abstract

Inversion polymorphisms have important phenotypic and evolutionary consequences in humans. Two different methodologies have been used to infer inversions from SNP dense data, enabling the use of large cohorts for their study. One approach relies on the differences in linkage disequilibrium across breakpoints; the other one captures the internal haplotype groups that tag the inversion status of chromosomes. In this article, we assessed the convergence of the two methods in the detection of 20 human inversions that have been reported in the literature. The methods converged in four inversions including inv-8p23, for which we studied its association with low-BMI in American children. Using a novel haplotype tagging method with control on inversion ancestry, we computed the frequency of inv-8p23 in two American cohorts and observed inversion haplotype admixture. Accounting for haplotype ancestry, we found that the European inverted allele in children carries a recessive risk of underweight, validated in an independent Spanish cohort (combined: OR= 2.00, *P* = 0.001). While the footprints of inversions on SNP data are complex, we show that systematic analyses, such as convergence of different methods and controlling for ancestry, can reveal the contribution of inversions to the ancestral composition of populations and to the heritability of human disease.

## INTRODUCTION

Inversion polymorphisms have been extensively studied in model organisms like the common fruit fly; however, they remain to be fully characterized in humans. An inverted allele is a specific DNA interval that runs backward with respect to a reference genome. They are bi-allelic loci with non-inverted and inverted status. Suppression of recombination when heterozygous promotes haplotype divergence within the region, contributing to segregation distortion, sex-chromosome evolution and reproductive isolation (1). Despite such evolutionary importance, a comprehensive map of sub-microscopic inversions in humans remains to be determined because of the lack of specific laboratory techniques to detect unknown events. Large-scale genotyping of known inversions is also difficult with established techniques like FISH (fluorescence *in situ* hybridization), due to cost and complexity of the genomic regions where inversions typically occur. invFest, a database with all reported inversions in the human genome, has been recently compiled (2). The compendium includes 85 experimentally validated inversions, 20 greater than 0.2 Mb and few with the inversion status of HapMap individuals. Inversions are cataloged as validated if directly observed by a laboratory technique such as FISH. The database also contains over 1100 predictions derived from paired end mapping (PEM).

Using genome-wide single nucleotide polymorphism (SNP) data, two other previous studies have also suggested that there could be hundreds of such mutations to be found in humans (3,4). Each study is based on different footprints of inversions on nucleotide variability, and therefore their derived algorithms provide complementary signals that indicate toward the presence of common inversion polymorphisms in population samples. Analysis of SNP genotypes has the important advantage that GWAS data can be used to study inversions in large cohorts. Given that experimental studies are still largely limited in the number of individuals and mutation events, these methods can also be used in conjunction to guide the discovery of unknown inversion polymorphisms. While it has been recognized the need of a clear strategy on how to include inversions in genome-wide association studies (5); until now, only three inversions observed by experimental techniques, namely inv-17q21, inv-8p23 and inv-16p11, have been studied with SNP dense data (6–8). The use of SNPs has allowed the characterization of the inversions’ global frequencies, evolutionary histories and contributions to phenotypic traits, gene expression and recombination (6,9–10). Nonetheless, the effect of inversions on SNPs can depend on segment size, history and population substructure, all of which can have a different impact on the methodology used to determine subject status. So far, only inv-16p11 has been simultaneously tested with the two bioinformatics approaches available to date (6).

One set of methods, which can identify up to 33% of predictions made by PEM, relies on the changes in linkage disequilibrium (LD) across breakpoints (3,11–12). The second set is based on the identification of haplotype groups supported by the suppression of recombination within the inverted segment (4,7,13). Such haplotype clustering has been shown to predict 2040 events, 169 of which overlapped with the 517 non-redundant inversions of the database of genomic variants (DVG). Scarce experimental genotyping of inversions does not yet allow the establishment of a benchmark method. On known inversions, however, convergence of these two approaches can inform of the sensitivity of the predictions and list the inversions that are amenable to study with SNP data. In addition, convergence of the methods on one unknown prediction can motivate its experimental study. In this work, we first aimed at studying the convergence of LD and haplotype methods to detect the 20 regions (>0.2 Mb) where inversion polymorphisms have been experimentally observed. For testing the convergence of LD- and haplotype-based methods we applied inveRsion (3) and invClust, respectively. invClust is a new haplotype method that we developed here. We used the HapMap III individuals to report the inversion frequencies in diverse populations and, in the available cases, test the convergence against inversion genotypes obtained from FISH and PEM predictions in previous studies.

As inv-8p23 is one of the inversions where both methods converged, we aimed to infer its status on individuals from an American population sample and test its association with phenotypic traits. Americans are stratified by ancestry and a significant proportion has mixed origins. Previous studies have split the data into ancestries for the analysis of global samples without admixture (4,7). However, for population samples with possible admixture between inversion haplotypes, this may not be suitable, in particular if the variability between ancestries is large. We thus applied invClust controlling for haplotype ancestry in order to recover the inversion genotype structure in the data. In relation to phenotypes, the 8p23 inversion is a *cis*-element that regulates the expression of *PPP1R3B*, which is a causal gene for low lipid levels in blood (14,15). Also within the inverted region in 8p23.1, an obesity study in the Hispanic population of American children has identified an SNP into the promoter region of *RPL1* associated with levels of cystathionine in blood (16). Patients with cystathionine beta synthase deficiency are characterized by low body mass index (BMI). Therefore, we assessed the association of inv-8p23 with low BMI in American children.

## MATERIALS AND METHODS

inveRsion is an algorithm that detects the changes of LD at the breakpoints (3). It computes the LD between contiguous blocks of SNPs that flank a potential breakpoint and tests if the expected pattern is likely to have been disrupted by the presence of an inversion. As such, inveRsion uses blocks of ∼10 SNPs flanking the breakpoints. On the other hand, invClust is a method based on all the SNPs inside the complete inverted segment that tests the existence of extended haplotypes, which may be sustained by the suppression of recombination due to the inversion. While invClust uses more SNPs, inveRsion is helped by SNPs outside the inversion. Therefore, the corpuses of observations of each method, for the signals they search, are essentially different.

### inveRsion

This method is based on differences in LD between SNP blocks across inversion breakpoints. Scanning the genome with predefined window sizes allows the search for inversion signals without previous knowledge of the breakpoints. A positive signal is given by the difference of Bayes Information Criterion (BIC) which, if greater than zero, indicates that the chromosomes of some individuals between the breakpoints tested are more likely to be inverted than not.

### invClust

In the context of haplotype tagging methods, we developed invClust. Previous studies have shown how dimensionality reduction analyses of the SNPs in the inverted region can be used to determine the inversion genotypes of individuals (4,7,13). Such methods apply statistical measures of the distance between inverted and non-inverted chromosomes. If the distance, e.g. genetic variability, between inversion status is larger than the distance within the same status then the first eigen-variables of the distance matrix can be used to cluster individuals into inversion homozygotes (I/I), standard homozygotes (NI/NI) and heterozygotes (I/NI). An inversion signal in this approach is therefore the identification of such a three-cluster pattern in the first components of a multidimensional scaling (MDS), or principal component, analysis. We developed a method to detect possible inversion genotype clustering in the data, based on a mixture model that includes particular constraints on the data as previously observed. These are Hardy Weinberg Equilibrium and equidistance between the three genotype clusters (4,7). The model therefore reduces the degrees of freedom and is more adjusted to the expected imprint of inversions on SNPs. In addition, it allows control on haplotype ancestry when necessary. This is particular important in the cases of population samples with possible admixture of ancestral haplotypes.

The model proposed in invClust comprises two components. The first one classifies the inversion genotypes of individuals with a similar haplotype origin, and the second one clusters individuals into ancestries. As a dimensionality reduction method, we use MDS analysis (7), although principal component analysis (PCA) can also be used (4). For inversion genotype classifications that need control of haplotype ancestry, we perform an MDS analysis on the entire sample and introduce a variable in the mixture model that classifies individuals according to ancestry (see Supplementary Methods).

### Data

We called the inversion genotypes on a number of data sets. We used the HapMap III genotypes downloaded from www.hapmap.org in the PLINK format (release 2 build 36). We were granted permission from dbGAP (http://www.ncbi.nlm.nih.gov/gap) to download the genotypes and clinical information from SNP Health Asthma Resource project (SHARP project accession number phs000166.v2.p1) and the Study of the Genetic Causes of Complex Pediatric Disorders (eMerge project accession number phs000490). The SHARP and eMerge projects contain genotype information of 3230 and 2480 American individuals from the general population, respectively. These two data sets were used to perform and validate the inversion calls. See dbGap accession number links for further information about study designs. The phenotypic association with the 8p23 inversion was assessed using 1532 children belonging to the SHARP study with available genotypes and normalized BMI (BMIz variable phv00070943.v1.p1). The association was validated using a Spanish sample of 906 children genotyped as part of the INfancia y Medio Ambiente birth cohort (INMA). Genotyping in INMA was performed using the Human Omni1 array and BMI was available for 758 children at 2 and 4 years of age.

### Detection and genotype call of known inversions

From the invFest database, we selected 20 experimentally validated inversions that are greater than 0.2 Mb (Supplementary Table S1), the detectability limit of inveRsion. Smaller inversions were not analyzed with invClust since, for such cases, extended haplotypes can naturally occur without the support of an inversion polymorphism. We run invClust within the breakpoints reported in the database to test the existence of a three-genotype cluster in the data, e.g. a positive inversion signal given by the algorithm. On the regions where clustering by invClust was possible, we run inveRsion to test whether this second algorithm detected LD differences between breakpoints.

For some experimentally validated observations, invFest offers the reported inversion genotypes of different HapMap individuals obtained by FISH, tagging methods and PEM, among others. We used the FISH and PEM available genotypes to assess the accuracy of invClust and inveRsion, when positive signals were found. For the HapMap trios we also tested the Mendelian inheritance of the inferred inversion status.

## RESULTS

### invClust and inveRsion convergence in HapMap individuals

From the invFest database (2), we selected 20 inversions (Table 1), all experimentally validated and greater than 0.2 Mb. Six of these are mutation events that have been observed by experimental techniques like FISH. We performed an MDS analysis of the SNPs within each of these segments for the entire HapMap III data and observed, from the first five MDS components, that three-genotype clustering is identifiable using the first two components. We then ran invClust on those components for each separate ancestry. For inversions in 17q12, 7q11 and 3q29 additional initial conditions were taken on the direction of highest variance. We found 10 inversions with three-cluster patterns in at least one of the HapMap populations, labeled as detected in Table 1. From the 10 remaining inversions that that did not show any genotype clustering, we observed that three inversions had less than 11 SNPs in the inverted region and no reliable inferences could be made, other six inversions have experimental support on only one individual. On inversion with high frequency and large number of SNPs (inv-15q13) showed strong demographic clustering and no inversion state structure.

**Table 1.** Twenty experimentally validated inversions tested with invClust

Band	Num snps	Validated	Detected
10p11-10q21	8215	3/23500	No
12p13-12p11	12353	1/1	No
12q23-12q24	11842	1/1	No
15q11-15q12	2665	4/44	No
4p16	3370	2/2	No
8p23	2956	95/100	Yes
7q11	563	17/305	Yes
15q13	735	11/27	No
3q29	833	2/19	Yes
9q21.33	1229	1/1	No
9q21.11	11	1/1	No
17q12	770	2/19	Yes
15q24	500	1/16	Yes
7p22	352	4/25	Yes
17q21	305	598/2767	Yes
1q12	2	1/1	No
12q24	220	1/1	Yes
16p11	76	11/14	Yes
9q21	2	1/1	No
17p11	36	1/7	Yes

invClust was run between reported coordinates (Supplementary Table S1) and containing the corresponding number of SNPs in the HapMap genotypes (second column). The third column shows the experimental support for each inversion as reported in invFest with the number of inverted chromosomes over the total number of individuals tested. The last column displays whether the segment presented a three-genotype clustering detected by invClust.

**Figure 1.**
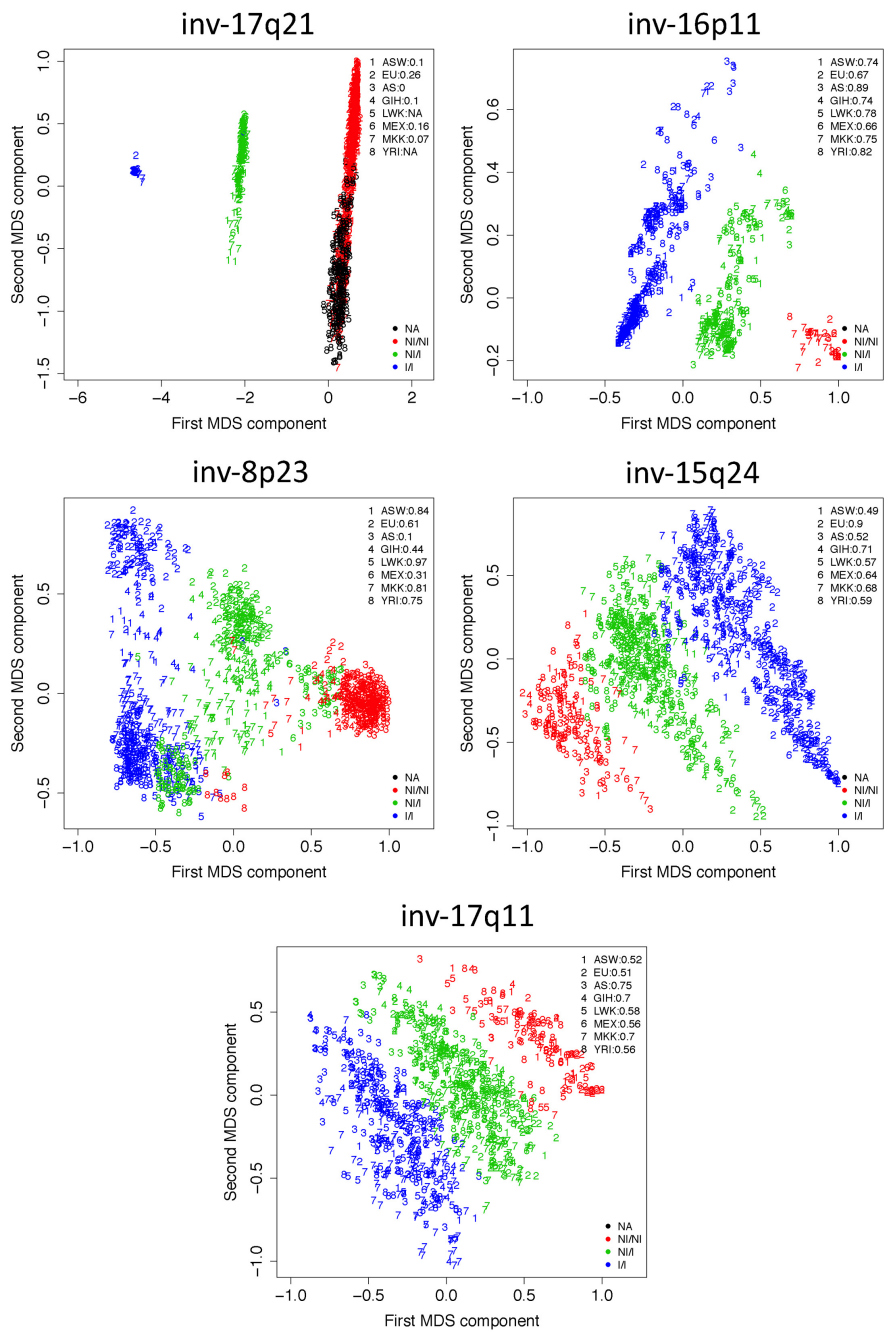
Inversion detection by invClust on five known inversions: three clusters are clearly observed for different HapMap III populations, corresponding to NI/NI: non-inverted homozygous; NI/I: inverted-heterozygous; and I/I: inverted-homozygous. Population frequencies are reported on the top right corner. European populations CEU and TSI are grouped into EU, and CHB, JPT and CHD into AS. Note that for inv-17q21 (top left) no signal was detected within YRI and MKK. Only relative comparisons with other populations indicate that their inversion frequencies are null.

On the 10 detected signals, we extracted the inversion status of individuals and tested Mendelian inheritance in the available trios. We found high consistency (99%) in the inheritance of the inversion haplotype groups identified in five cases, see Table 2 and Figure 1. Among them are inv-17q21, inv-16p11 and inv-8p23, which also showed accuracy greater than 95% with inversion genotypes determined by FISH or tagging variants. Accuracy given by PEM data on eight individuals was lower, particularly for 8p23 (25%). The two other inversions with high Mendelian inheritance, inv-15q24 and inv-17q11, had some support from FISH (72%) and PEM (87%) data, respectively. Sensitivity and specificity on the classification of inversion status were also computed from the accuracy against FISH data (Supplementary Table S2). Other five inversions with clustering detected by invClust but low inheritance accuracy (<99%) are shown in Supplementary Figure S1.

**Figure 2.**
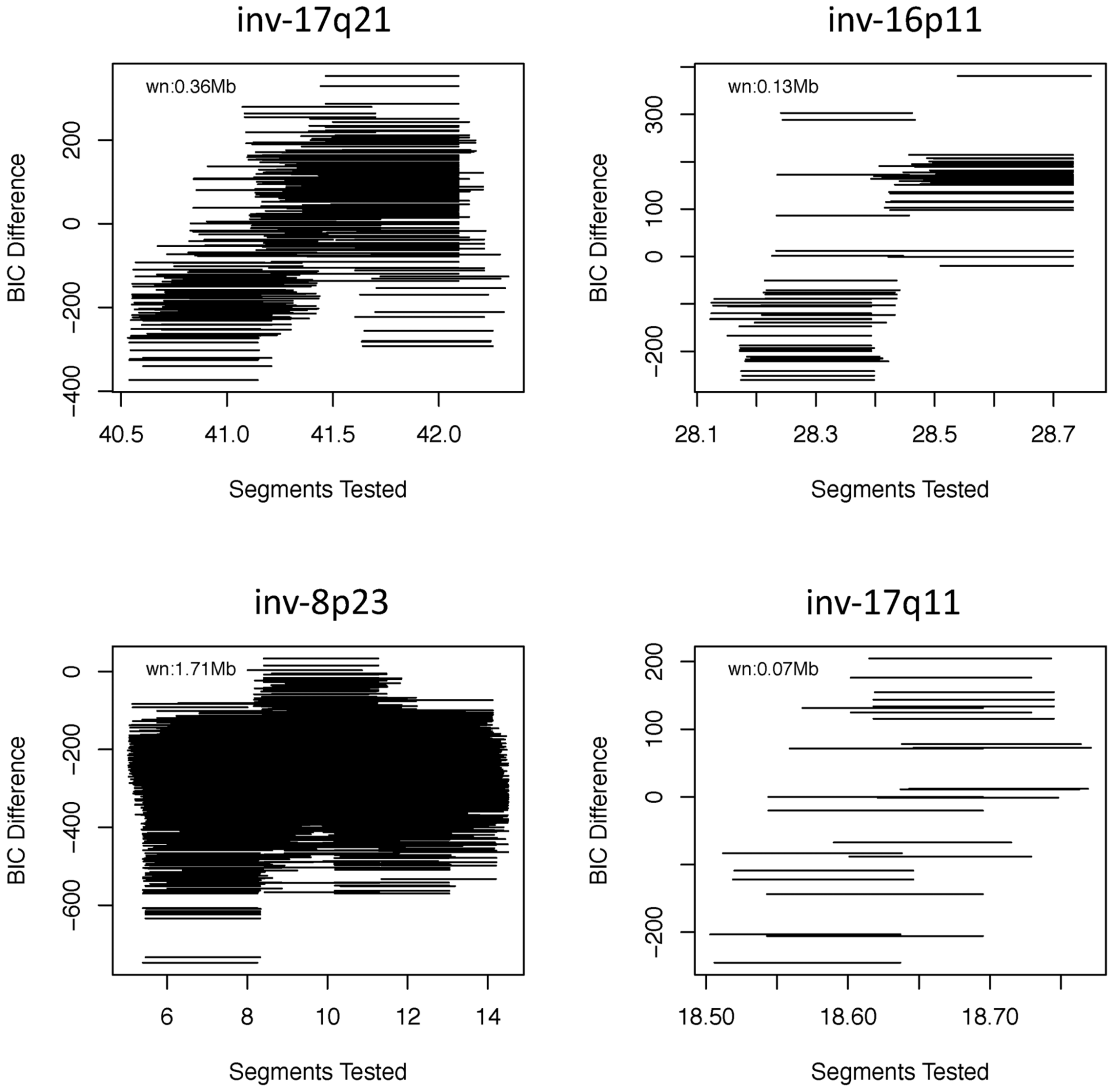
Inversion detection by inveRsion on four known inversions: the segments show the breakpoints across which LD differences are compared within an inversion model of the data. The segments are windows of size *wn*. BIC differences greater than zero indicate that the inversion model (individuals with some inverted segments) is preferred over the non-inversion model (no individual with inverted segment). The inversion model consists of the detection of subjects for whom the internal block between breakpoints should be inverted to account for unexpected large LD at distant points.

We then ran inveRsion around the 10 regions in Table 2 on the Utah residents with Northern and Western European ancestry from the CEPH collection (CEU) individuals only, as LD patterns can substantially change between ancestries. We scan the regions looking for inversion signals with six different window sizes ranging from 40 to 90% of the expected inverted segment (Figure 2). We found signals supporting the inversion model (BIC > 0), in inv-17q21, inv-16p11, inv-8p23 and inv-17p11; around the expected breakpoints. Finally, we compared the prediction of inveRsion with invClust on the 165 inversion genotypes of the CEUs and observed accuracies greater than 90%.

**Table 2.** Positive detection of 10 known polymorphic inversions from SNP data

Validated inversions				invClust prediction			
Band	chr	LBP	RBP	Men. (*n* = 128)	FISH/tag (*n*)	PEM (*n* = 8)	inveRsion (*n* = 165)
17q21	17	40928986	42139672	100%	99.9%(1668)	87%	98%
16p11	16	28256775	28695952	100%	100%(14)	87%	90%
8p23	8	6909899	12617968	99%	95%(118)	25%	94%
15q24	15	72140039	73384192	99%	72%(24)	37%	-
17q11	17	18442024	18692134	99%	NA	87%	91%
17q12	17	31799963	33389579	98%	66%(24)	25%	-
7p22	7	5899796	6839043	97%	NA	62%	-
12q24	12	130339370	130724947	97%	100%(1)	-	-
7q11	7	71967070	74995983	96%	NA	25%	-
3q29	3	196830755	198878785	94%	60%(24)	75%	-

invClust was run between the reported coordinates (from first to fourth columns). Accuracy of invClust classification of inversion genotypes was assessed against Mendelian inheritance (Men.) and genotyping made by 1) FISH or tagging methods (FISH/tag), 2) PEM predictions (PEM) and 3) inveRsion. inveRsion was run on the CEU population with different window sizes on the 10 inversions and accuracy was assessed against invClust predictions. Four signals were detected supporting the inversion model. *n* refers to the number of individuals used in each comparison. Both algorithms were run with their default parameters.

### Inversion call of inv-8p23 in an American population sample

**Figure 3.**
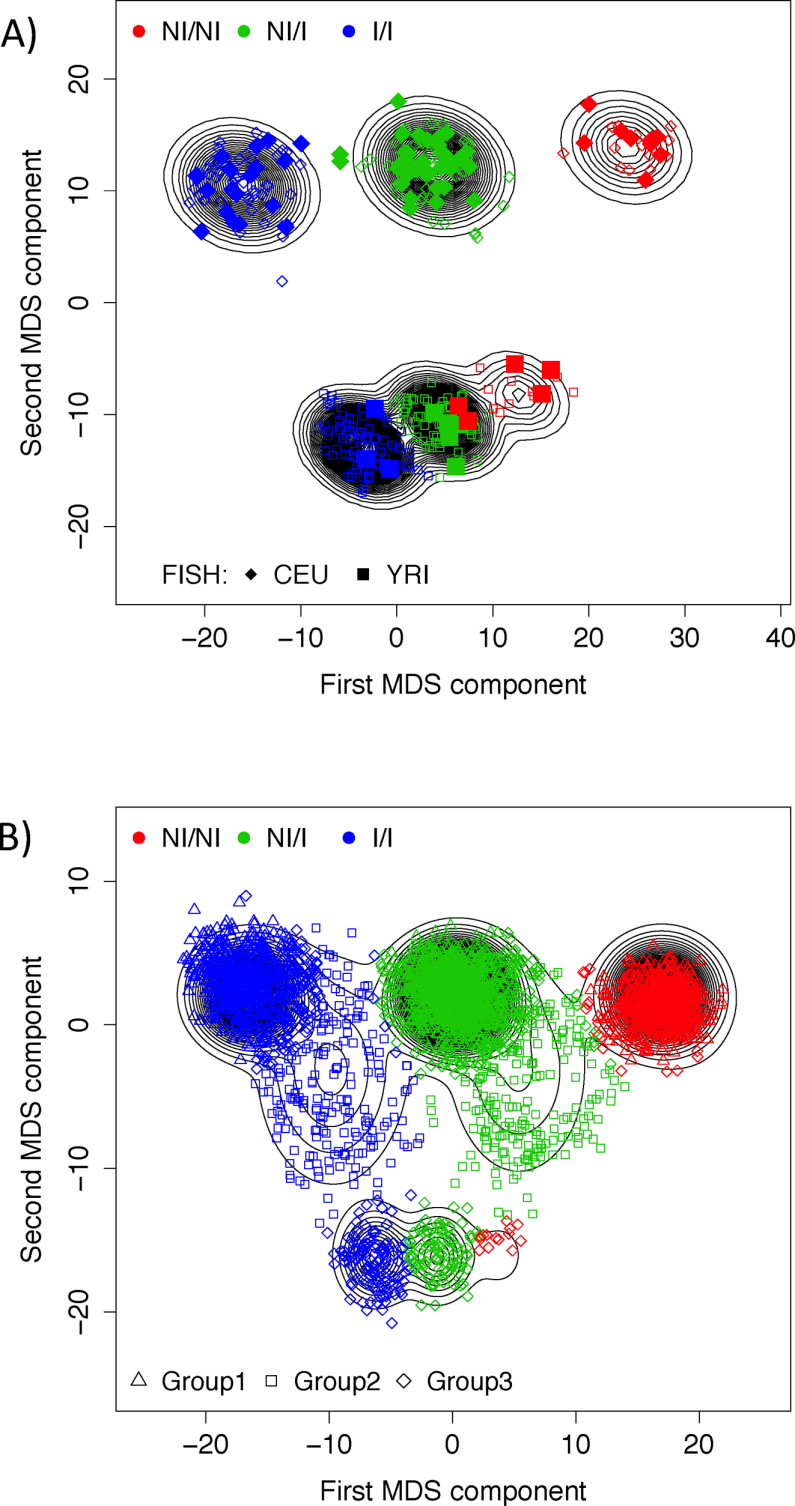
Inv-8p23 detection by invClust on samples with different haplotype ancestry: (**A**) simultaneous detection of inversion genotypes on the CEU and YRI individuals. Inferences were validated with existent FISH data. (**B**) Simultaneous inference of inversion genotypes on 3230 American individuals from the general population. The algorithm detected European (group1: triangles), Mixed (group2: squares) and African (group3: diamond) haplotype ancestries, together with the inversion genotype clusters.

As inv-8p23 has consistent signals between methods, we aimed to characterized it in a population sample of Americans and assess its association with a hypothesized phenotype. Since Americans have diverse genetic origins, we first selected the CEU and Yoruba in Ibadan, Nigeria (YRI) HapMap individuals to assess the performance of invClust in classifying simultaneously inversion genotypes from European and African haplotype ancestries. We fitted the model using the second component of the MDS analysis in the inverted region as our ancestry informative variable (*x* in Supplementary Methods). Figure 3A shows the fitted model with the inferred inversion genotypes. We validated these results with published genotypes determined by FISH (7). We correctly predicted 72 of the 74 genotypes. The two misclassified heterozygous subjects are in the YRI sample and close to the area of maximum overlap between the genotype groups NI/I and NI/NI. The accuracy limit is mainly given by the multivariate approach to infer inversion genotypes rather than a specific clustering method. Unreported experimental error cannot be ruled out either.

We then analyzed a general population sample of Americans. We performed an MDS analysis on the entire sample of 3230 American children (SHARP) and found that the second MDS component showed three possible haplotype ancestries. Therefore, we applied the genotype call algorithm, using the second MDS component to classify the ancestries, in a similar manner to the previous analysis for the joint set of CEU and YRI individuals (Figure 3B). The algorithm classified the individuals into three ancestry groups; group 1: European; group 2: Mixed; group 3: African. As a result, all self-reported ethnicities were stratified by inversion ancestry (European-Americans: 90% European and 10% Mixed; African-Americans: 4% European, 38% Mixed and 58% African; Hispanics: 85% European, 14% Mixed and 1% African). The substantial split of the European and African-American individuals into their inversion haplotype ancestries was not detected with a genome-wide ancestry classification, as measured with a genome-wide PCA (Supplementary Figure S2). Regarding inversion genotypes, we found inverted allele frequencies of 48, 73 and 74% for European, Mixed and African haplotype ancestries. Using a comparable American sample, we ran the same model on the eMerge cohort (*N* = 2480) and validated the cluster pattern and the inverted allele frequencies (European: 53%; Mixed: 71%; African: 74%). We identified a similar clustering of haplotype ancestries (Supplementary Figure S3).

**Figure 4.**
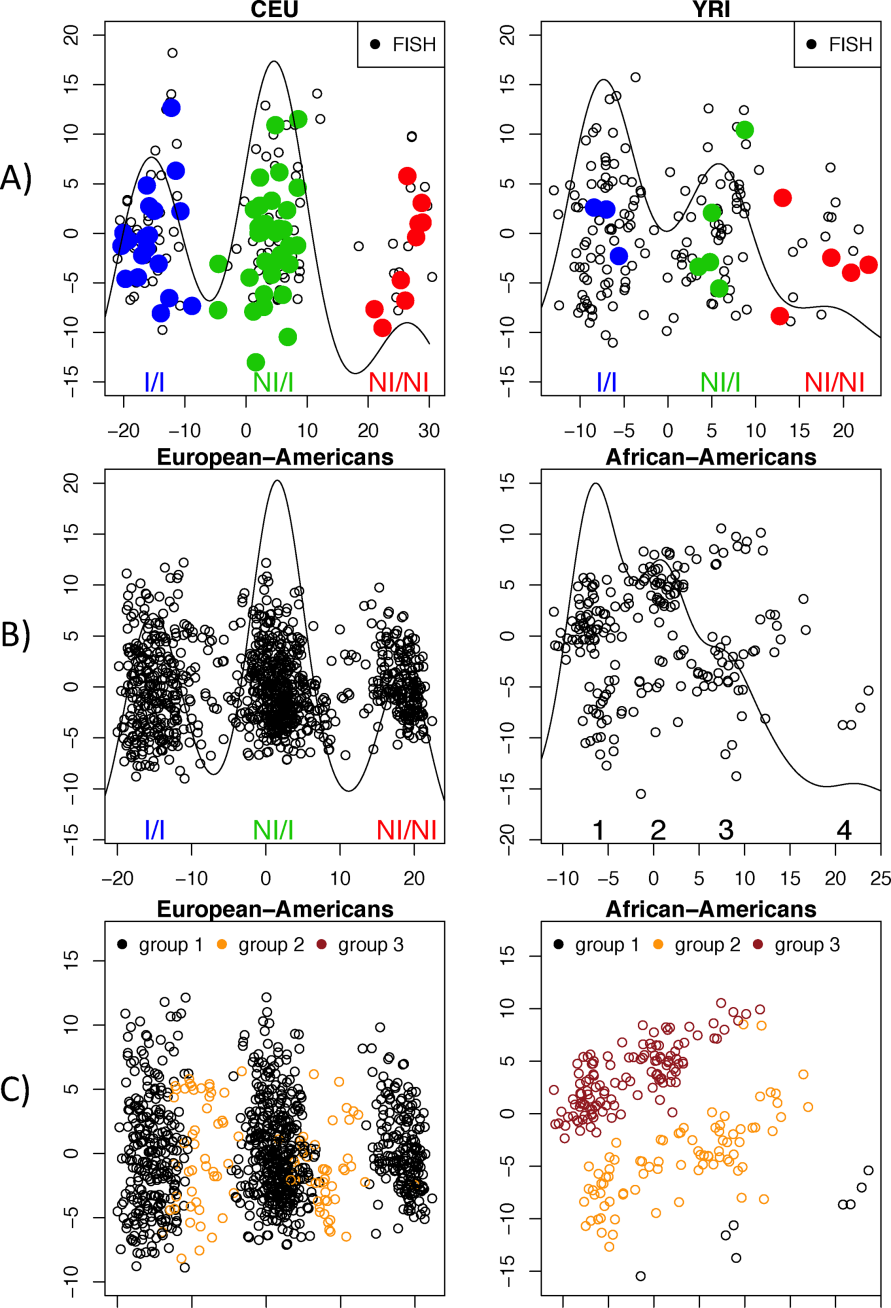
First two MDS components of SNPs in the 8p23-inverted region for individuals from different ancestries: (**A**) independent detection of inversion genotypes of CEU and YRI individuals. FISH observations are shown with filled circles, validating the inversion genotype clusters. (**B**) Genotype clustering on self-reported European-American and African-Americans from the SHARP study. While in the European-Americans the inversion genotypes are clearly differentiated, in self-reported African-Americans, inference of genotype clusters cannot be reliably performed as more than three clusters are obtained in both MDS components. (**C**) Ancestry haplotype control performed by invClust on the entire data set shows that European and African-Americans are stratified by three ancestry groups.

We observed that the model recovered the inversion genotype structure of the data if we split the individuals into haplotype ancestries and not into those that have been self-reported or inferred by genome-wide PCA. We early noted that inversion detection on each separate ethnicity showed additional clustering structure, which does not correspond to the three-cluster pattern observed for CEUs and YRIs (Figure 4A and B). The classification by invClust, controlling for haplotype ancestry, later revealed that all ethnic groups are stratified by European, Mixed and African haplotype ancestries (groups 1, 2 and 3 in Figure 4C), in addition to the inversion genotypes. Such haplotype stratification confounded inversion classification by a previous algorithm, PFIDO (7), exclusively developed to genotype inv-8p23 in samples from reference populations. PFIDO applied on African-Americans with reference on YRIs detected only two clusters in the data, ignoring the sub-clustering (Supplementary Figure S4). For self-reported European-Americans, we observed that Mixed individuals (group 2) fall between the genotype clusters (Figure 4C). Therefore, European-Americans have a greater uncertainty in genotype calling if haplotype ancestry is not controlled for.

### Association between inv-8p23 and BMI

We used the inversion genotype call from the previous analysis to perform association test with BMI. From the SHARP study, we had BMI data for 1532 children, with a mean age of 4 years. We fit regression models between BMI and inversion genotypes adjusting for the first two genome-wide PCA components, to account for genome-wide population stratification. We classified individuals into underweight children (BMI < 5th-percentile) and controls (BMI > 5th-percentile), which corresponds to the standard definition of underweight by the World Health Organization. We found a significant association for the recessive model (Table 3). Thus, inverted homozygous had a risk of low BMI (OR = 1.67, *P*-value = 0.03). Other genetic models were not-significant nor associations with BMI as continuous variable.

**Table 3.** Association between inv-8p23 and low BMI in children

		BMI > 5th-perc.	BMI < 5th-perc.	OR	95% CI	*P*-value
**SHARP**	**ALL**					
	Recessive					
	N/N-N/I	68.7%	57.1%	1		
	I/I	31.3%	42.9%	1.67	(1.03, 2.70)	0.03
	**European haplotype ancestry**					
	(inferred ancestry group 1)					
	Recessive					
	N/N-N/I	75.1%	60%	1		
	I/I	24.9%	40%	1.92	(1.11, 3.35)	0.02*
	**European-American**					
	(self-reported)					
	Recessive					
	N/N-N/I	68%	55.4%	1		
	I/I	32%	44.6%	1.71	(0.99, 2.95)	0.05
**INMA**	**ALL**					
	Recessive					
	N/N-N/I	66%	50%	1		
	I/I	34%	50%	2.10	(1.09, 4.08)	0.02

Top: association analyses between inv-8p23 genotypes and low BMI in the SHARP sample, adjusted for the first two genome-wide PCA components. While individuals with European haplotype ancestry have a significant risk for low BMI, self-reported European-Americans do not. The *P*-value with an asterisk is significant after correction for multiple genetic models. Bottom: association analysis between inv-8p23 and low BMI in the INMA sample. Combined effects between the two cohorts resulted in OR = 2.00, *P*-value = 0.001 and population attributable risk of 2.8%.

In order to determine the contribution of haplotype ancestries to the association, we performed stratified analyses. Stratification into the European haplotype ancestry revealed increments in the magnitude and significance of the association (OR = 1.92, *P*-value = 0.02). The result was significant after correcting for multiple comparisons for different genetic models (17). We also observed that stratification into self-reported European-Americans decreased the significance of the association (OR = 1.71, *P*-value = 0.057), consistent with our observation that up to 10% of individuals in this group may be misclassified as they are of Mixed haplotype ancestry (Figure 4C). Since, in addition, no evidence for associations was found for Mixed or African ancestries (Supplementary Table S3), these results suggested that the association could be specific to European haplotypes.

As misclassification of the associations could affect the results, we validated our findings using a sample of Caucasian Spanish children, where no haplotype ancestry stratification was observed (Supplementary Figure S5). The inversion frequency was 57%, consistent with the inversion's global distribution in Europeans. BMI was measured in 758 children at 2 and 4 years of age. We classified children with low BMI as those under the fifth percentile of the average BMI between the second and fourth year of age. We tested the recessive model, as it was the most significant in the previous analysis, and found that Spanish children who are inverted homozygous also have a significant risk of being underweight (OR = 2.1, *P*-value = 0.02). Therefore, associations between low BMI in children and inv-8p23 in two independent cohorts, INMA and SHARP, were similar in magnitude, had an increased combined significance (combined OR = 2.00, *P*-value = 0.001) and a substantial attributable risk of 2.8%.

## DISCUSSION

In this work, we tested the convergence of LD- and haplotype-based methods to assess the imprint of known inversion polymorphism on SNPs, in humans. The assessment was limited to long inversions that were previously discovered by direct experimental observations. The methods are expected to perform best in ancestral inversions with no or very low recurrence, where enough SNP variability is accumulated between the inverted and non-inverted states. We observed that the two approaches converged on three inversions previously studied with SNP data, namely inv-17q21, inv-16p11 and inv-8p23. An additional inversion in 17p11 also showed consistent signals between methods. While there are no FISH data to assess the true inversion genotyping of individuals for this inversion, PEM predictions on eight individuals disagreed on only one subject and Mendelian error was less than 1%. Therefore, further experimental characterization of this inversion calls for attention as well as association tests with hypothesized phenotypes. In addition, the convergence of both methods on the most studied inversions with SNPs encourages the experimental evaluation of unknown cases where both methods detect a positive signal.

LD and clustering methods are profuse in the detection of new inversions, as compared with more direct approaches such as PEM predictions. However, predicting inversions with PEM is also difficult in regions flanked by large segmental duplications, which is the case for all inversions observed with congruent signals. Here, we studied the sensitivity of both methods to detect experimentally validated inversions and justify association tests with phenotypes in large cohorts. We found that the LD method, inveRsion, has low sensitivity and only detected four inversion signals of the 10 cases detected by invClust. A possible source for the increment of power by invClust can come from the fact that a positive signal in this method is given by all the SNPs in the region while inveRsion only exploits SNPs at the breakpoints. In addition, as differences in LD across breakpoints could depend on the joint effects of ancestry similarity and time of the mutation (3), inveRsion should be more optimal for homogenous samples and old inversions. Consistent with this, we observed that inveRsion detects inversions for which the frequency of African ancestry is greater than that of European ancestry, with the only exception of inv-7p22, which we ruled out due to high Mendelian error.

Within the haplotype-based method, a detailed look into the MDS analysis of the inversions with low Mendelian error revealed a different SNP footprint on each of them. While inv-17q21 showed the archetypical three-cluster pattern across all HapMap III populations, which can be simulated with coalescent theory (4,18), inv-16p11 and inv-8p23 have additional sub-clusters. In the case of inv-16p11, we observed six clusters that have been previously shown to correspond to three haplotypes supported by the inversion (6). Here, we found that the additional structure of inv-8p23, on the other hand, arises from the ancestry differences, African and European, in inverted and non-inverted haplotypes. Our results thus indicate that inversions’ effect on the SNPs can be diverse, possibly affected by their specific evolutionary histories.

Interpretation of the MDS components in terms of sources of genetic divergence is particularly straightforward for inv-8p23, for which the first MDS component can be interpreted as divergence due to the inversion and the second as divergence due to ancestry. Under this context, it is interesting to note that inversion haplotypes for Africans appear less divergent than for Europeans, but further characterization of the haplotypes is needed to account for such difference. We observed that the first two MDS components were able to identify genotype clustering for most inversions; however, in general, state and ancestry could load on different components. Extension of the methods including more components should be considered to study improvements in the classification. In the case of inv-8p23, we saw that the haplotype ancestry could be introduced with the second MDS component. While this may also vary in other inversions, invClust allows ancestry information to be included independently of the MDS analysis (*x* variable in Supplementary Methods). invClust can also be used on other multivariate analyses, like principal or independent component analysis, that summarize the genetic variability in different ways. The method is flexible to accommodate the complexities of each case. Assessment of true subject classification for most of the inversions awaits further experimental work to establish the precise mapping between haplotype groups and inversions status.

Controlling for inversion origin in the calling of inv-8p23, we detected three inverted allele ancestries in two independent American samples. The inferred haplotype ancestries indicate that the total frequency of African (inverted and non-inverted) haplotypes is 77% in self-reported African-Americans, which is consistent with estimates showing that they are 72% African and 20% European (19). In addition, Hispanics have 8% of African haplotypes, which falls in the expected range (20). African ancestry of admixed individuals is then identifiable with inv-8p23, as the inverted haplotypes are long (∼4.5 Mb) and suppression of recombination is restricted to the same inversion status.

Taking into account haplotype ancestry, we found an unknown association between the 8p23 inversion allele and low BMI in children. The risk for underweight was restricted to inverted haplotypes of European ancestry. This observation was validated in an independent sample, where we observed a high and reproducible effect size for an allele with more than 50% frequency in Europeans. This is a novel and relevant association given the high population that is at attributable risk of 2.8%. While we successfully validated the association tests, the *P*-values in both the discovery and validation sets were significant but modest. Therefore, association tests of inv-8p23 with other related phenotypes and higher sample sizes are granted.

To our knowledge, this is the first time that a phenotypic association is performed for an inversion polymorphism in an admixed population. Our stratification analysis showed that inclusion of individuals with haplotype ancestries, rather than European, increased the uncertainty of the association. In particular, the association was reduced in self-reported European-Americans, where 10% had mixed haplotypes. This indicates that not controlling for haplotype ancestry can reduce the significance of the associations by misclassification of inversion genotypes or specificity to genetic origin, even if the population sample is split into self-reported ethnicities. Further studies are required to determine the impact of phenotypic variation by ancestry on the same inversion allele, particularly for the inversions that may be under selective pressures (6–8,21).

Overall, we show that inversion polymorphisms leave imprints in SNP data, which can be studied with bioinformatics methods. The different methodologies can shed light into the existence of unknown inversions and be used to study their effects on phenotypic traits, guiding future experimental efforts. While sequencing methods can also detect inversions (22), evolutionary and association studies in humans are largely dominated by SNP microarray data. Therefore, characterization of inversions with SNPs currently offers the most cost-effective method for their study in large cohorts. Not all inversions can be studied with SNPs, however; and more efforts are needed to map bioinformatics predictions with experimental genotyping.

## AVAILABILITY

The methods developed here have been implemented in an R package invClust, which can be downloaded from http://www.creal.cat/jrgonzalez/software.htm.

## SUPPLEMENTARY DATA

Supplementary Data are available at NAR Online.

SUPPLEMENTARY DATA

SUPPLEMENTARY DATA

## References

[1] Kirkpatrick M. (2010). How and why chromosome inversions evolve. PLoS Biol..

[2] Martínez-Fundichely A., Casillas S., Egea R., Ràmia M., Barbadilla A., Pantano L., Puig M., Cáceres M. (2014). InvFEST, a database integrating information of polymorphic inversions in the human genome. Nucleic Acids Res..

[3] Cáceres A., Sindi S.S., Raphael B.J., Cáceres M., González J.R. (2012). Identification of polymorphic inversions from genotypes. BMC Bioinformatics.

[4] Ma J., Amos C.I. (2012). Investigation of inversion polymorphisms in the human genome using principal components analysis. PLOS ONE.

[5] Feuk L. (2010). Inversion variants in the human genome: role in disease and genome architecture. Genome Med..

[6] González J.R., Cáceres A., Esko T., Cuscó I., Puig M., Esnaola M., Reina J., Siroux V., Bouzigon E., Nadif R. (2014). A common 16p11.2 inversion underlies the joint susceptibility to asthma and obesity. Am. J. Hum. Gen..

[7] Salm M.P., Horswell S.D., Hutchison C.E., Speedy H.E., Yang X., Liang L., Schadt E.E., Cookson W.O., Wierzbicki A.S., Naoumova R.P. (2012). The origin, global distribution, and functional impact of the human 8p23 inversion polymorphism. Genome Res..

[8] Stefansson H., Helgason A., Thorleifsson G., Steinthorsdottir V., Masson G., Barnard J., Baker A., Jonasdottir A., Ingason A., Gudnadottir V.G. (2005). A common inversion under selection in Europeans. Nat. Genet..

[9] deJong S., Chepelev I., Janson E., Strengman E., van denBerg L.H., Veldink J.H., Ophoff R.A. (2012). Common inversion polymorphism at 17q21. 31 affects expression of multiple genes in tissue-specific manner. BMC Genomics.

[10] Alves J.M., Chikhi L., Amorim A., Lopes A.M. (2014). The 8p23 inversion polymorphism determines local recombination heterogeneity across human populations. Genome Biol. Evol..

[11] Sindi S.S., Raphael B.J. (2010). Identification and frequency estimation of inversion polymorphisms from haplotype data. J. Comp. Biol..

[12] Bansal V., Bashir A., Bafna V. (2007). Evidence for large inversion polymorphisms in the human genome from HapMap data. Genome Res..

[13] Deng L., Zhang Y., Kang J., Liu T., Zhao H., Gao Y., Li C., Pan H., Tang X., Wang D. (2008). An unusual haplotype structure on human chromosome 8p23 derived from the inversion polymorphism. Hum. Mutat..

[14] Teslovich T.M., Musunuru K., Smith A.V., Edmondson A.C., Stylianou I.M., Koseki M., Pirruccello J.P., Ripatti S., Chasman D.I., Willer C.J. (2010). Biological, clinical and population relevance of 95 loci for blood lipids. Nature.

[15] Global Lipids Genetics Consortium (2013). Discovery and refinement of loci associated with lipid levels. Nat. Genet..

[16] Comuzzie A.G., Cole S.A., Laston S.L., Voruganti V.S., Haack K., Gibbs R.A., Butte N.F. (2012). Novel genetic loci identified for the pathophysiology of childhood obesity in the Hispanic population. PLOS ONE.

[17] González J.R., Carrasco J.L., Dudbridge F., Armengol L., Estivill X., Moreno V. (2008). Maximizing association statistics over genetic models. Genet. Epidemiol..

[18] O'Reilly P.F., Coin L.J., Hoggart C.J. (2010). invertFREGENE: software for simulating inversions in population genetic data. Bioinformatics.

[19] Murray T., Beaty T.H., Mathias R.A., Rafaels N., Grant A.V., Faruque M.U., Watson H.R., Ruczinski I., Dunston G.M., Barnes K.C. (2010). African and non-African admixture components in African Americans and an African Caribbean population. Genet. Epidemiol..

[20] Galanter J.M., Fernandez-Lopez J.C., Gignoux C.R., Barnholtz-Sloan J., Fernandez-Rozadilla C., Via M., Hidalgo-Miranda A., Contreras A.V., Figueroa L.U., Raska P. (2012). Development of a panel of genome-wide ancestry informative markers to study admixture throughout the Americas. PLoS Genet..

[21] Steinberg K.M., Antonacci F., Sudmant P.H., Kidd J.M., Campbell C.D., Vives L., Malig M., Scheinfeldt L., Beggs W., Ibrahim M. (2012). Structural diversity and African origin of the 17q21. 31 inversion polymorphism. Nat. Genet..

[22] Kidd J.M., Cooper G.M., Donahue W.F., Hayden H.S., Sampas N., Graves T., Hansen N., Teague B., Alkan C., Antonacci F. (2008). Mapping and sequencing of structural variation from eight human genomes. Nature.

