# Supplementary material for: Following the footprints of polymorphic inversions on SNP data: from detection to association tests
Source: Nucleic Acids Res. 2015 Feb 11;43(8):e53. doi: 10.1093/nar/gkv073 (PMC4417146; doi:10.1093/nar/gkv073)
Supplement: SUPPLEMENTARY DATA [file supp_gkv073_supplementary-1.pdf]

Supplementary Methods: `invClust`

We assume that the likelihood to observe one subject with the first two MDS components $\boldsymbol{y} = (y_1, y_2)$ of the SNPs in the inverted region is proportional to the three component mixture

$$\begin{aligned}
L(\Theta; \boldsymbol{y}) \propto f(\Theta; \boldsymbol{y}) &= \sum_{r=1}^{3} p_r^2 f_r(\theta_r; \boldsymbol{y}) \\
f_1(\theta_1; \boldsymbol{y}) &= e^{-\frac{1}{2}(\boldsymbol{y}-\boldsymbol{\mu_1})^t \boldsymbol{\Sigma}^{-1}(\boldsymbol{y}-\boldsymbol{\mu_1})} \\
f_2(\theta_2; \boldsymbol{y}) &= e^{-\frac{1}{2}(\boldsymbol{y}-\boldsymbol{\mu_2})^t \boldsymbol{\Sigma}^{-1}(\boldsymbol{y}-\boldsymbol{\mu_2})} \\
f_3(\theta_3; \boldsymbol{y}) &= e^{-\frac{1}{2}(\boldsymbol{y}-\boldsymbol{\mu_3})^t \boldsymbol{\Sigma}^{-1}(\boldsymbol{y}-\boldsymbol{\mu_3})}, \qquad (1)
\end{aligned}$$

where $r = 1, 2, 3$ denotes the three inversion genotypes (1: non inverted homozygous, 2: heterozygous and 3: inverted homozygous), with frequenies p_1^2, p_2^2 and p_3^2 and means $\boldsymbol{\mu_1} = (\mu_{11}, \mu_{12})^t$, $\boldsymbol{\mu_2} = (\mu_{21}, \mu_{22})^t$ and $\boldsymbol{\mu_3} = (\mu_{31}, \mu_{32})^t$. $\boldsymbol{\Sigma}$ is the symmetric (2×2) covariance matrix between y_1 and y_2, which we assume equal for all three genotype groups. Θ refers to all parameters and θ_r to those of the mixing genotype component r. In our model, p_1 and p_3 are the alelle frequencies that correspond to the homozygous frequencies p_1^2 and p_3^2. The model is constrained to distributions satisfying HWE and the equisitance condition

$$\begin{aligned}
p_1^2 + 2p_1p_3 + p_3^3 &= 1 \qquad (2) \\
\boldsymbol{\mu}_2 &= \frac{1}{2}(\boldsymbol{\mu}_1 + \boldsymbol{\mu}_3). \qquad (3)
\end{aligned}$$

The first equation implies $p_2^2 = 2p_1p_3$ for the frequency of the inverted heterozygous. Based on this model, the probability of observing a genotype 1, 2 or 3 for a subject with MDS components $\boldsymbol{y}$ is

$$\begin{aligned}
\omega_1(\boldsymbol{y}; \Theta') &= \frac{p'^2_1 f_1(\theta_1; \boldsymbol{y})}{f(\Theta'; \boldsymbol{y})} \\
\omega_2(\boldsymbol{y}; \Theta') &= \frac{2p'_1 p'_3 f_2(\theta_1, \theta_3; \boldsymbol{y})}{f(\Theta'; \boldsymbol{y})} \\
\omega_3(\boldsymbol{y}; \Theta') &= \frac{p'^2_3 f_3(\theta_3; \boldsymbol{y})}{f(\Theta'; \boldsymbol{y})}. \qquad (4)
\end{aligned}$$

The prime denotes the current value of the model parameters that we will update with the expectation maximization (EM) algorithm. The maximization step follows from the optimization, at all the model parameters (Θ), of

the function

$$Q(\Theta,\Theta') = \sum_{j=1}^{n}\sum_{r=1}^{3}\omega_{rj}\log\{|\mathbf{\Sigma}|^{-1/2}p_r^2 f_r(\theta_r;\boldsymbol{y_j})\}, \tag{5}$$

which is written in terms of probabilities of inversion status r for each subject j: $\omega_{rj} = \omega_r(\boldsymbol{y}_j;\Theta')$ and $j = 1...n$. Maximization with respect to p_1 and p_3 is conditioned to equation 2, which can be incuded as a Lagrange multiplier λ in the solution of $\partial_{p_1,p_3}Q(\Theta,\Theta') = 0$. Using equations (1), then such conditional maximization of Q translates into solving

$$\partial_{p_1,p_3}\{\sum_{j=1}^{n}(2\omega_{1j}+\omega_{2j})\log p_1 + (2\omega_{3j}+\omega_{2j})\log p_3 \\ -\lambda(p_1^2+2p_1p_3+p_3^2-1)\} = 0 \tag{6}$$

for p_1, p_3 and λ. We thus obtain the solution of the allele frequencies

$$p_1 = \frac{1}{2}\frac{\sum_j(2\omega_{1j}+\omega_{2j})}{\sum_{rj}\omega_{rj}} \tag{7}$$

$$p_3 = \frac{1}{2}\frac{\sum_j(2\omega_{3j}+\omega_{2j})}{\sum_{rj}\omega_{rj}}. \tag{8}$$

Maximization of Q with respect to the cluster means is simplified by changing to variables $\boldsymbol{\mu}_a = \boldsymbol{\mu}_1 + \boldsymbol{\mu}_3$ and $\boldsymbol{\mu}_b = \boldsymbol{\mu}_1 - \boldsymbol{\mu}_3$. Therefore, after deriving with respect to $\boldsymbol{\mu}_a$ and $\boldsymbol{\mu}_b$ and equating to zero, we find

$$\boldsymbol{\mu}_a = \frac{\sum_j \boldsymbol{y}_j(\omega_{1j}-\omega_{3j})\sum_k(\omega_{3k}-\omega_{1k}) + \sum_{rj}\boldsymbol{y}_j\omega_{rj}\sum_k(\omega_{1j}+\omega_{3j})}{\sum_j(\omega_{1j}-\omega_{3j})\sum_k(\omega_{3k}-\omega_{1k}) + \sum_{rj}\omega_{rj}\sum_k(\omega_{1j}+\omega_{3j})}$$

$$\boldsymbol{\mu}_b = \frac{\sum_j(\boldsymbol{y}_j-\boldsymbol{\mu}_a)(\omega_{1j}-\omega_{3j})}{\sum_j(\omega_{1j}+\omega_{3j})}. \tag{9}$$

In the case of clustering the inversion genotypes of one single population, these equations can be further simplified with $\omega_{1j}+\omega_{2j}+\omega_{3j} = 1$. However, such relationship is not held in the general case of dealing with diverse inversion ancestries. Therefore, we leave (7)-(9) in their most explicit form for the purpose of the most general model. Finally the maximization with respect to covariance parameters follows the usual multivariate mixture modelling

$$\mathbf{\Sigma} = \frac{\sum_{rj}\omega_j(\boldsymbol{y}_j-\boldsymbol{\mu}_r)(\boldsymbol{y}_j-\boldsymbol{\mu}_r)^t}{\sum_{rj}\omega_{rj}} \tag{10}$$

where $\boldsymbol{\mu}_1 = (\boldsymbol{\mu}_a + \boldsymbol{\mu}_b)/2$, $\boldsymbol{\mu}_2 = \boldsymbol{\mu}_a/2$ and $\boldsymbol{\mu}_3 = (\boldsymbol{\mu}_a - \boldsymbol{\mu}_b)/2$. Equations (7)-(10) constitute the updating of the model parameters, which can then be used to re-estimate (4) and repeat the process until the parameter estimates converge to machine precision. We are then interested in final values of ω_{rj} whose maximum value over r gives us the genotype of subject j; that is the genotype call for each individual in the sample.

As EM estimations can fall into local minima of the likelihood, we tested different initial conditions, from all possible combinations of

$$\begin{aligned} \boldsymbol{\mu}_1 &= (Q_{0.75}[y_1], 0) \\ \boldsymbol{\mu}_3 &= (Q_{0.75}[y_1] + 2\mathrm{Sd}[y_1], \max[y_2]) \\ \boldsymbol{\Sigma} &= \mathbf{1}_{2\times 2} \\ p_1 &= 0.5, 0.9 \\ p_3 &= 0.5, 0.1 \end{aligned} \tag{11}$$

where $Q_{0.75}[y_1]$ is the 75% quantile of y_1, $\mathrm{Sd}[y_1]$ is its standard deviation and y was normalized by $max(y_1, y_2)$. The final model was selected with the smallest Bayesian information criterion, including a non-inversion model resulting from the Gaussian distribution of the data. We validated these results by comparing the frequency of the inversion for each HapMap population as illustrated in Table 1. The table shows the frequency of the inversion polymorphism according to published findings, and according to the results of the separate subsample analysis.

Controlling for different haplotype ancestries

For inversion genotype classifications that need control of haplotype ancestry, we first perform a MDS analysis on the entire sample and introduce a new variable that classifies individuals according to ancestry. Let us assume a general ancestry information variable x. Therefore the likelihood of observing an individual with inversion x and $\boldsymbol{y}$ is given as before but with the additional ancestry mixture detected by x

$$\begin{aligned} L(\Theta; x, \boldsymbol{y}) &\propto f(\Theta; x, \boldsymbol{y}) \qquad (12) \\ &= \sum_{g=1}^{l} \sum_{r=1}^{3} \pi_g p_{rg}^2 f_{rg}(\theta_{rg}; \boldsymbol{y}) e^{-\frac{1}{2}(x-\mu_g)^2/\sigma_g^2}. \end{aligned}$$

Component definitions follow equations (1), with the exception that all previous parameters depend on the ancestry clustering $g = 1...l$, i.e θ_r is now θ_{rg}.

The inversion ancestry groups are modelled by Gaussian distributions with mixing parameters π_g, giving the probabilities that one subject is observed with value x. Given the observations $\boldsymbol{y}$ and x, the probability of observing a subject in genotype r and group g is

$$\omega_{rg}(x, \boldsymbol{y}; \Theta') = \frac{\pi'_g {p'}^2_{rg} f_{rg}(\theta'_{rg}; \boldsymbol{y}) e^{-\frac{1}{2}(x-\mu'_g)^2/{\sigma'}^2_g}}{f(\Theta'; x, \boldsymbol{y})}. \tag{13}$$

The new estimates of the model parameters follow from the maximization of

$$\begin{aligned} Q(\Theta, \Theta') = & \sum_{j=1}^{n} \sum_{g=1}^{l} \sum_{r=1}^{3} \omega_{rgj} \log\{ \frac{|\boldsymbol{\Sigma}_g|^{-1/2}}{\sigma_g} \pi_g p^2_{rg} \\ & f_r(\theta_{rg}; \boldsymbol{y_j}) e^{-\frac{1}{2}(x-\mu_g)^2/\sigma^2_g} \} \end{aligned} \tag{14}$$

where $\omega_{rgj} = \omega_{rg}(x, \boldsymbol{y}_j; \Theta')$. The estimates for the genotype mixing components p_{1r}, p_{3r}, $\boldsymbol{\mu}^1_g$ $\boldsymbol{\mu}^3_g$ and $\boldsymbol{\Sigma}_g$ follow from equations (7)-(10) for each subgroup g, while the subpopulation clustering parameters π_g, μ_g and σ_g are the usual estimates of a mixture of g components

$$\pi_g = \frac{1}{n} \sum_{rj} \omega_{rgj} \tag{15}$$

$$\mu_g = \frac{\sum_{rj} x_j \omega_{rgj}}{\sum_{rj} \omega_{rgj}} \tag{16}$$

$$\sigma^2_g = \frac{\sum_{rj} \omega_{rgj} (x_j - \mu_g)^2}{\sum_{rj} \omega_{rgj}}. \tag{17}$$

The inversion genotype for each subject was obtained by the marginal probability $\omega_{ij} = \sum_g \omega_{1gj}$.

Supplementary Plots S1-S5 and Tables S1-S3

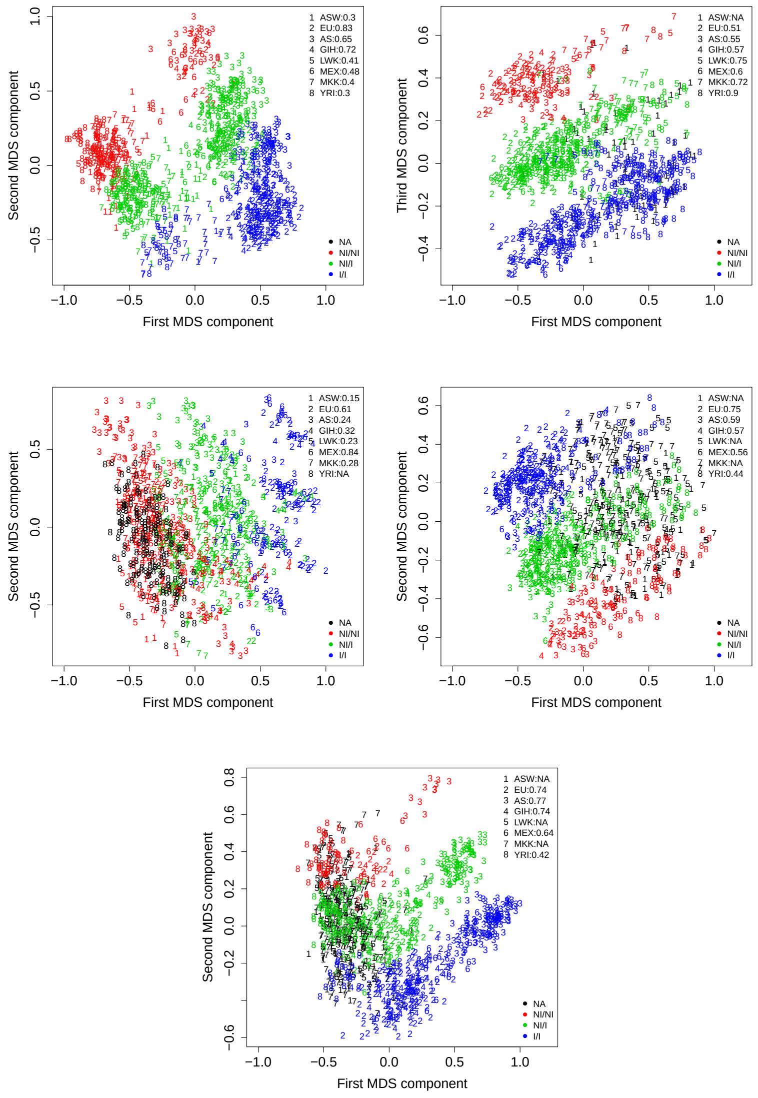

Figure S1. Inversion detection by `invClust` on additional known inversions: From left to righ, top to bottom: 17q12, 7p22, 12q24, 7q11 and 3q29.

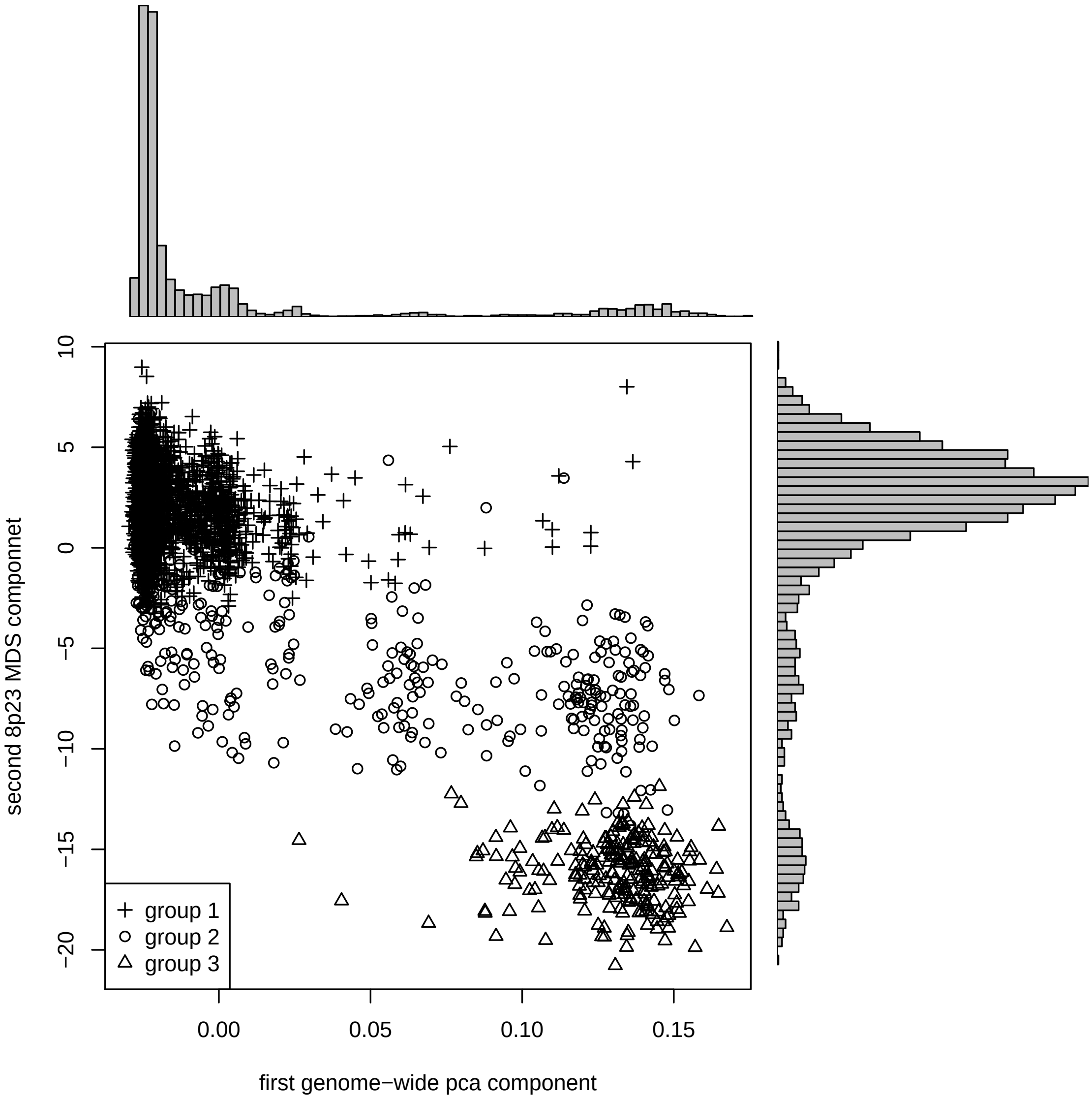

Figure S2. The second MDS component of SNPs within 8p23 against the first genome-wide PCA component. The PCA component preserves most of the African-American and European-Hispanics in two separate sub-population clusters while the second MDS component splits the data into three ancestry groups. Other PCA components did not show such split.

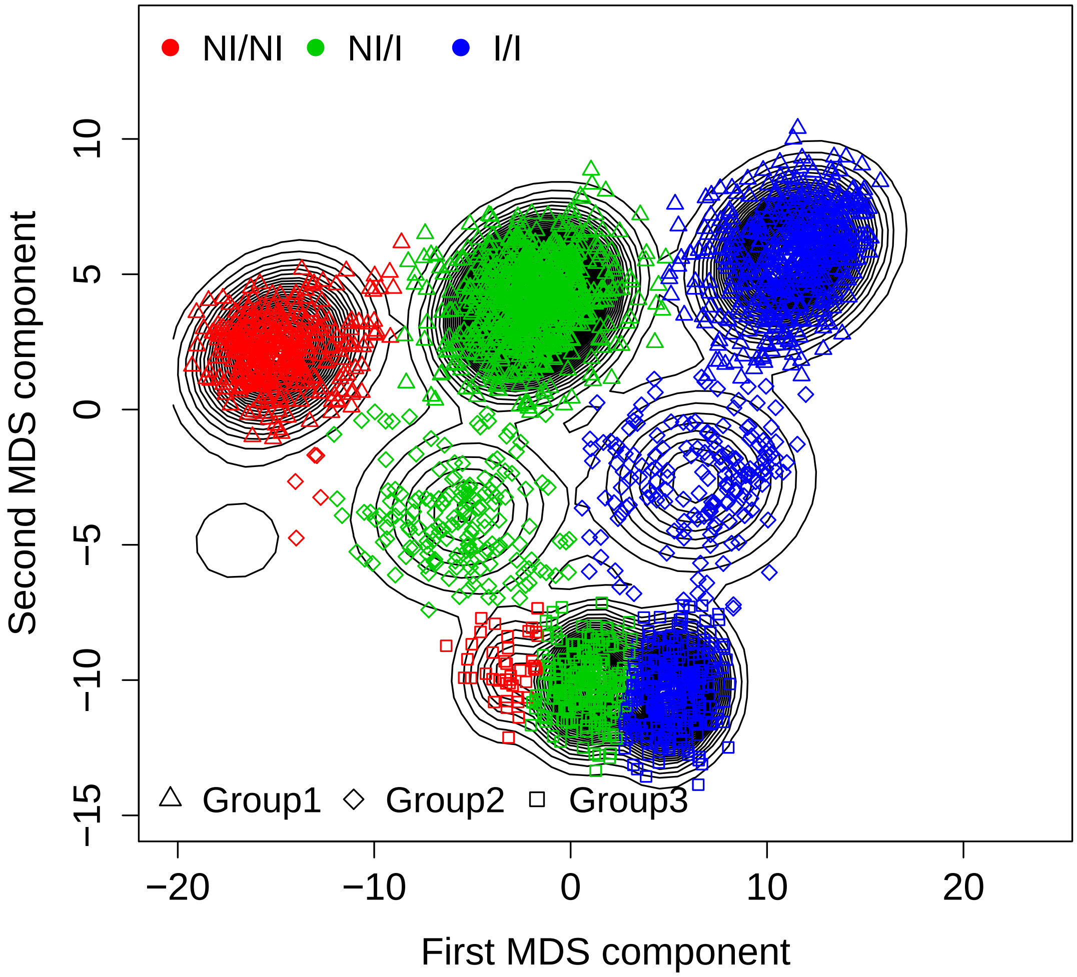

Figure S3. Full mixture model conditioning on HWE and accounting for population stratification for 2480 American children in the eMerge sample. The classification provides inversion frequencies consitent with those of the SHARP sample. It also validates the split of self reported African-Americans into two groups.

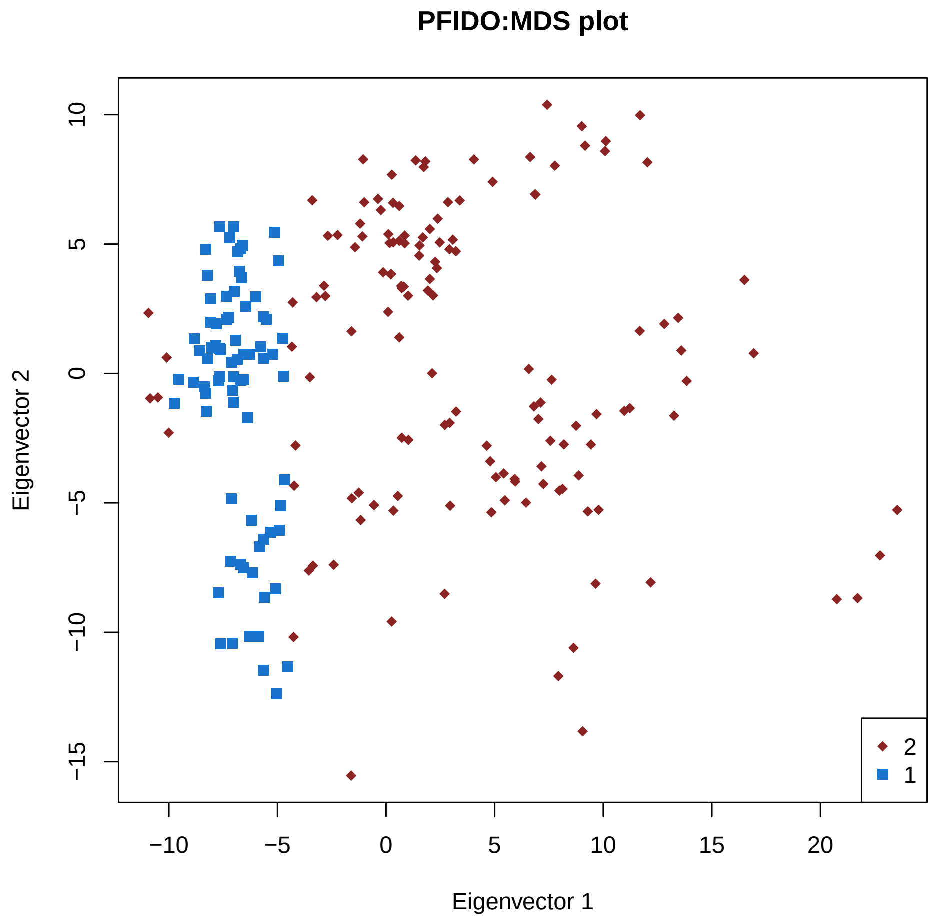

Figure S4. Inference of 8p23 inversion on the African-American individuals of the SHARP sample using PFIDO which does not control for haplotype ancestry.

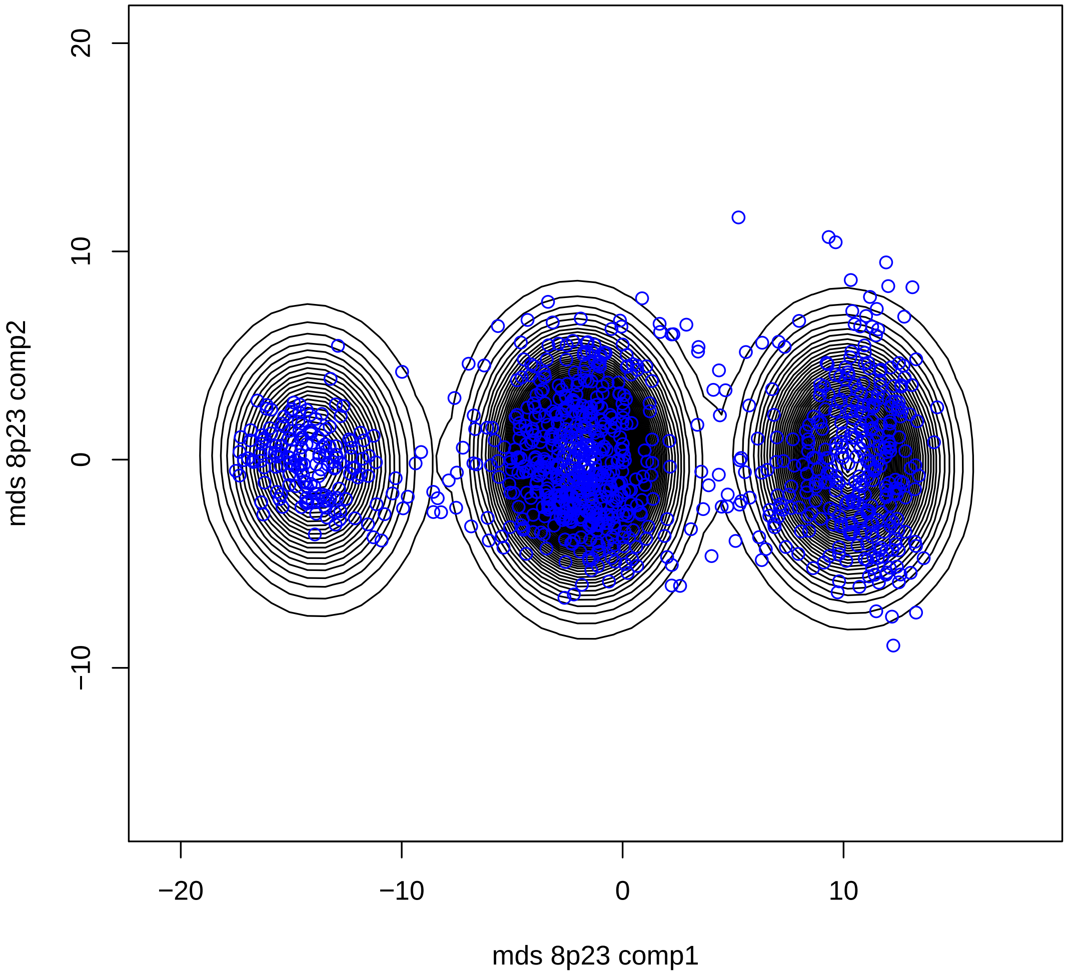

Figure S5. Inversion call on 906 Spanish children of the INMA cohort using the first two MDS scaling of SNPs in the 8p23 inverted region. The figure shows three clear clusters corresponding to each inversion genotypes with no population stratification.

chr	LBP	RBP
10	37148086	59748194
12	9508936	31097166
12	102899673	124371107
15	20646359	26896735
4	3866029	9373835
8	6909899	12617968
7	71967070	74995983
15	28157404	30687000
3	196830755	198878785
9	85694926	87647892
9	66189745	67910343
17	31799963	33389579
15	72140039	73384192
7	5899796	6839043
17	40928986	42139672
1	141476058	141913529
12	130339370	130724947
16	28256775	28695952
9	67994583	68298521
17	18442024	18692134

Table S1. List of 20 inversions selected from `invFest` with inverted segment $> 0.2Mb$ and for which experimental support has been reported.

band	chr	LBP	RBP	accuracy(n)	sensitivity	specificity
17q21	17	40928986	42139672	99.9%(1668)	100%	99%
16p11	16	28256775	28695952	100%(14)	100%	100%
8p23	8	6909899	12617968	95%(118)	95%	95%
15q24	15	72140039	73384192	72%(24)	100%	72%
17q12	17	31799963	33389579	66%(24)	66%	64%
12q24	12	130339370	130724947	100%(1)	100%	-
3q29	3	196830755	198878785	60%(24)	50%	61%

Table S2. **Sensitivity/Specificity of `invClust` on inversion call against FISH data**. Estimates obtained from accuracy calulations in table 1 of manuscript. *n* stands for number of subjects with FISH data as reported on `invFest`.

	BMIz>5th-perc.	BMIz<5th-perc.	OR	95%CI	p-val
**Mixed haplotype ancestry**					
Recessive					
N/N-N/I	50.4%	55.6%	1.00		
I/I	49.6%	44.4%	0.86	(0.22, 3.38)	0.82
**African haplotype ancestry**					
Recessive					
N/N-N/I	47.5%	37.5%	1.00		
I/I	52.5%	62.5%	1.40	(0.31, 6.27)	0.65
**African-American**					
Recessive					
N/N-N/I	51%	37.5%	1.00		
I/I	49%	62.5%	1.75	(0.40, 7.56)	0.44
**Hispanics**					
Recessive					
N/N-N/I	87.6%	66.7%	1.00		
I/I	12.4%	33.3%	1.65	(0.12, 23.34)	0.71
**Others**					
Recessive					
N/N-N/I	68.1%	80%	1.00		
I/I	31.9%	20%	0.81	(0.11, 5.87)	0.82

Table S3. Top: association analyses between low BMI and 8p23 inversion genotypes in the SHARP sample for self-reported ethnic groups (African-American, Hispanics and Others) and for inferred inversion ancestries (Mixed and African).
