# Supplementary material for: Following the footprints of polymorphic inversions on SNP data: from detection to association tests
Source: Nucleic Acids Res. 2015 Feb 11;43(8):e53. doi: 10.1093/nar/gkv073 (PMC4417146; doi:10.1093/nar/gkv073)
Supplement: SUPPLEMENTARY DATA [file supp_43_8_e53_v2_index.html]

Following the footprints of polymorphic inversions on SNP data: from detection to association tests — SUPPLEMENTARY DATA 

# Following the footprints of polymorphic inversions on SNP data: from detection to association tests

## SUPPLEMENTARY DATA

**Files in this Data Supplement:**

- SUPPLEMENTARY DATA
